# Sedation Modulates Frontotemporal Predictive Coding Circuits and the Double Surprise Acceleration Effect

**DOI:** 10.1093/cercor/bhaa071

**Published:** 2020-05-19

**Authors:** Adrien Witon, Amirali Shirazibehehsti, Jennifer Cooke, Alberto Aviles, Ram Adapa, David K Menon, Srivas Chennu, Tristan Bekinschtein, Jose David Lopez, Vladimir Litvak, Ling Li, Karl Friston, Howard Bowman

**Affiliations:** 1 School of Computing, University of Kent, Kent CT2 7NF, UK; 2 Center for Neuroprosthetics, EPFL, Sion 1951, Switzerland; 3 East Kent Hospitals University NHS Foundation Trust, Kent & Canterbury Hospital, Canterbury CT1 3NG, UK; 4 Institute of Psychiatry, Psychology & Neuroscience, King’s College London, London SE5 8AF, UK; 5 School of Psychology, University of Birmingham, Birmingham B15 2TT, UK; 6 Division of Anaesthesia, Box 97, Cambridge Biomedical Campus, University of Cambridge, Cambridge CB2 0QQ, UK; 7 Department of Psychology, University of Cambridge, Cambridge CB2 3EB, UK; 8 Electronic Engineering program, Universidad de Antioquia, Ciudad Universitaria, Medellín 1226, Colombia; 9 Wellcome Centre for Neuroimaging, University College London, London WC1N 3AR, UK

**Keywords:** predictive coding, global workspace, source inversion, EEG analysis

## Abstract

Two important theories in cognitive neuroscience are predictive coding (PC) and the global workspace (GW) theory. A key research task is to understand how these two theories relate to one another, and particularly, how the brain transitions from a predictive early state to the eventual engagement of a brain-scale state (the GW). To address this question, we present a source-localization of EEG responses evoked by the local-global task—an experimental paradigm that engages a predictive hierarchy, which encompasses the GW. The results of our source reconstruction suggest three phases of processing. The first phase involves the sensory (here auditory) regions of the superior temporal lobe and predicts sensory regularities over a short timeframe (as per the local effect). The third phase is brain-scale, involving inferior frontal, as well as inferior and superior parietal regions, consistent with a global neuronal workspace (GNW; as per the global effect). Crucially, our analysis suggests that there is an intermediate (second) phase, involving modulatory interactions between inferior frontal and superior temporal regions. Furthermore, sedation with propofol reduces modulatory interactions in the second phase. This selective effect is consistent with a PC explanation of sedation, with propofol acting on descending predictions of the precision of prediction errors; thereby constraining access to the GNW.

## Introduction

Two important theories in current cognitive neuroscience are predictive coding (PC; Rao and Ballard 1999; Friston 2010) and global neuronal workspace (GNW) theory (Dehaene and Changeux 2011). The former emphasizes forward and backward exchanges along sensory processing and higher level pathways, with forward connections carrying prediction errors, and backward connections conveying predictions. In contrast, the latter emphasizes a distinct mode of processing—the global workspace (GW)—which has the character of a sustained brain-scale state, into which there is a sharp transition—described as ignition (Dehaene et al. 2006).

Indeed, King et al. (2014) argued for the existence of two distinct modes of processing; the first restricted to sensory areas and the latter, the GNW. In addition, they have suggested that these two modes are experimentally engaged by the local-global task. This is an auditory deviance task in which tones can be unexpected at two different levels of regularity. The first level, which generates the (so-called) local effect, reflects regularity at a short temporal frame of reference, that is, repeated “tones.” In contrast, the second level, which generates the (so-called) global effect, reflects regularity at a longer temporal frame; that is, repeated sequences of tones.

In this way, the local-global task engages a PC hierarchy, with multiple levels at which prediction errors could arise, in much the same vein as proposed by PC (Friston et al. 2006). GW theory would, though, additionally propose the existence of a spatially broad and temporally extended “brain-scale” state at the top (or center) of this hierarchy, which would be associated with the global effect. Key to reconciling PC and GW theory is to understand how the transition from a predictive early stage (disclosed by the local effect) to engagement of a brain-scale state (disclosed by the global effect) is mediated. Understanding this transition—or ignition—is the objective of the work presented here. In other words, how do lower levels of a processing hierarchy come to engage higher levels—and what are the underlying neurophysiological mechanisms? In this respect, our work contrasts interestingly with recent research with related objectives (for example, Chao et al. 2018).

PC postulates relatively fast coordinated exchanges up and down a multilayered hierarchy, while the GNW argues for longer term, “metastable” states; clearly, how one transitions between these two is of fundamental interest. Additionally, in terms of similarities, both PC and the GW assume an underlying hierarchy of neuronal message passing or what, from a Bayesian perspective, could be considered belief propagation. Furthermore, both associate higher (or deeper) levels with perceptual synthesis at longer timeframes.

Under PC, the influence of ascending prediction errors depends upon their precision (i.e., reliability or inverse variance). This has often been cast in terms of attentional selection, where ascending prediction errors that are afforded greater precision are selected to have a greater influence on belief updating at higher hierarchical levels (Auksztulewicz and Friston 2015; Kanai et al. 2015; Parr and Friston 2017). On this view, ignition—or a transition to global processing throughout the depth of cortical hierarchies—rests upon the top-down control of the precision of bottom-up signals (i.e., prediction errors). This gracefully relates attentional processing to conscious content that gains access to the GW, while referring to a measurable aspect of neuronal processing; namely, the modulation of excitability of synaptic gain (which precision controls) of neuronal populations reporting prediction errors.

One clear hypothesis that follows from PC, and has informed our study, is that a transition from local to global processing would be accompanied by descending modulation from higher cortical levels to lower cortical levels. To inform this hypothesis empirically, we use the local global paradigm—in conjunction with source localization (to establish the hierarchical level of neuronal activity)—and characterize responses evoked by (local and global) violations as a function of peristimulus time. To test the hypothesis that evoked responses reflect modulatory interactions between high and low cortical levels, we crossed the local global paradigm with an inhibitor of neuromodulation; namely, propofol. Propofol has been previously shown to modulate extrinsic hierarchical connections, that is, involving across-brain sources (Boly et al. 2012; Gómez et al. 2013), and indeed, to do this within the context of the local-global task (Uhrig et al. 2014, 2016; Nourski et al. 2018). Furthermore, the use of an anesthetic offers the opportunity for construct validation, in the sense that it reduces levels of conscious processing, of the sort associated with the GW (Uhrig et al. 2016).

Our previous work with the local-global task (focusing on responses in sensor space) suggested that the transition from local to global processing is not temporally or functionally as sharp as one might have thought (Shirazibeheshti et al. 2018). Indeed, our findings could be interpreted as evidence for a transitional phase between the local and global responses. This evidence rests on an interaction between the local and global conditions (Shirazibeheshti et al. 2018). In other words, early responses to global violations depend on the presence of a local violation. Alternatively, the late responses to local violations depend upon a global violation. This interaction appears to be driven by changes in responsiveness during the positive rebound to the N1 and mismatch negativity (MMN), that is, during the P3a window (~200 to ~ 400 ms poststimulus onset). The key aspect of this change in responsiveness is a coincidence of surprise; that is, a combination of local and global deviants induces an acceleration of the P3, an effect we have called the double-surprise acceleration effect (Shirazibeheshti et al. 2018) (The term accelerate is primarily used metaphorically, although, the advanced P3 component that we identified in [Shirazibeheshti et al. 2018] [see Figure 5 L x G panels in that paper] does in fact exhibit an acceleration in the mathematical sense. That is, the tangent to the curve [the velocity] changes rapidly with time, meaning that the rate of the rate of change with time [i.e., the acceleration] is bigger in the advanced P3 [the LDGD condition].). Furthermore, manipulation of awareness (through sedation) was found to modulate this interaction.

In this paper, we extend these findings. As just discussed, we have established a transition between local and global processing phases, temporally and functionally. Here, we characterize this transition neurophysiologically—in terms of hierarchical processing in the brain. That is, we ask the following question: can we—in respect of brain areas—observe a transition from a localized (low-level) prediction error to a (high-level) global ignition? In particular, can we neurophysiologically identify the phase transition, which we propose is an additional transitional stage? A further key question is the role of awareness in this hierarchical processing. In particular, as Dehaene et al. would argue, is it only the late, global processing that engenders states of awareness (Dehaene 2011) (see also, Uhrig et al. 2016)?

To answer these questions, we report a Multiple Sparse Priors (MSP; Friston et al. 2008) source localization of the local-global task. This enabled us to characterize the neurophysiological trajectory of neuronal responses as they propagate from rapidly changing early responses restricted to sensory areas (for us in auditory cortices) to slowly changing late (c.f., metastable) responses (involving temporal, frontal and parietal areas). Our key finding was that the transition between these two phases—sensory-bound to global—involves a transient engagement of the superior temporal–inferior frontal network. Furthermore, the interaction between local and global violations on responses in this network is attenuated by propofol sedation.

## Methods

### Participants

Originally, 22 neurologically healthy adults were included in the study, but two recordings were lost due to technical issues, leaving 20 participants (9 male; 11 female) (mean age = 30.85; SD = 10.98).

#### Experimental Design

**Figure 1:**
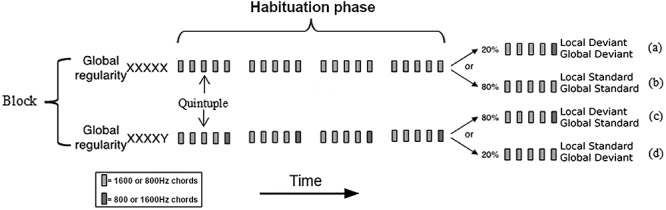
Local-global auditory task design from (Bekinschtein et al. 2009).

The local-global auditory oddball task, devised by Bekinschtein et al. (2009), was used to characterize differences between local and global effects after healthy sedation and subsequent recovery. As shown in Figure 1, local regularity was established using sequences of five tones, or quintuples, where the last tone may or may not vary from the preceding four tones (local deviant [LD] vs. local standard [LS], respectively). Global regularity was established as the most frequently presented quintuple type within a block, either LS (all five tones the same) or LD (different last tone). Thus, violations in global regularity were expressed by the presentation of a quintuple that differed from the frequently presented type in any block. To ensure global regularity was established, a habituation period of 20–30 quintuples was presented at the beginning of the block. After the habituation phase, the ratio between the standard and deviant quintuples was set to 80/20. This created four conditions: (1) local standard/global standard (LSGS), (2) local deviant/global standard (LDGS), (3) local standard/global deviant (LSGD), and (4) local deviant/global deviant (LDGD) (see Fig. 1*a*–*d*). Quintuples comprised 5 tones of 50 ms duration each, presented via headphones, with an intensity of 70 dB and an SOA of 150 ms. All tones were synthesized with 7 ms rise and 7 ms fall times. Participants were asked to count the number of global deviants they heard during both sedation and recovery phases of the study as an incidental task to reduce fluctuations in attentional set.

#### Sedation

During surgery or procedures for diagnosing medical conditions, it is common to take the patient to a sedative plane (as opposed to general anesthesia); also for pain control, it is common to use propofol to relax the patient or take him/her/them to the point of sleep. In sedation research studies, the state is defined either by the target concentration in blood (light, medium or moderate sedation; light, medium or deep anesthesia) and/or a clinical responsiveness scale like the Ramsay (https://www.sciencedirect.com/topics/medicine-and-dentistry/ramsay-sedation-scale). However, in the study analyzed in this paper, the sedation states are defined in an even more detailed manner, by exact concentration of propofol in blood in each state and by RTs and response misses in a behavioral task, as described for this experiment in Chennu et al. (2016b).

In the experiment we are analyzing, we knew that the target concentrations induced light to moderate sedation, where participants range from mild changes in relaxation to mostly unresponsive, but easily “arousable,” since these dose-responses had been defined already, as in Stamatakis et al. (2010), Adapa et al. (2014), Barttfeld et al. (2015), and David et al. (2007).

Specifically, we knew the behavioral and drug in blood pattern, enabling us to place participants around “the verge of unconsciousness.” The specifics of how we manage to maintain participants in this state with such small doses are elaborated in Absalom et al. (2009).

The local-global task was presented on two occasions; once during either mild (half of participants) or moderate (the other half) sedation and once 20 min later, when participants were considered to be in recovery (i.e., no longer sedated). Sedation in this study induced a heavily relaxed but behaviorally responsive state. All participants were tested both under sedation and subsequently in recovery, creating a repeated measures design. Each experimental run began with an awake baseline period lasting 25–30 min followed by a target-controlled infusion of propofol (Marsh et al. 1991), administered via a computerized syringe driver (Alaris Asena PK, Carefusion). Three blood plasma levels were taken –0.6 μg/mL (mild sedation), 1.2 μg/mL (moderate sedation), and after recovery from sedation. A period of 10 min was allowed for equilibration of calculated and actual plasma propofol concentrations before cognitive tests commenced. Following cessation of infusion, plasma propofol concentration exponentially declined toward zero and approached zero in 15 min leading up to behavioral recovery. In light of this, the recovery condition started 20 min after cessation of sedation.

All procedures were conducted in accordance with the Declaration of Helsinki. The participants provided written informed consent and were healthy controls. Ethical approval for testing healthy controls was obtained from the Cambridgeshire two Regional Ethics Committee.

#### EEG Recording

During preprocessing, two patients were excluded due to artifacts; therefore, 18 participants were taken forward for analysis. Participants were asked to close their eyes during data collection to avoid eye artifacts in the data. EEG data were collected on two occasions: during sedation and then recovery. A Net Amps 300 amplifier (Electrical Geodesic Inc.) with a high-density cap of 129 channels was used for data acquisition, and preprocessed data were obtained using custom Matlab scripts based on EEGLab. EEG time-series were recorded in microvolts (μV), with a sampling frequency of 250 Hz, and referenced to vertex (channel Cz). After recording, the data were segmented from –200 ms before the first tone in a quintuple until 1296 ms after that tone. Bad channels (those that crossed a 200uVs threshold) were interpolated. The remaining trials were rereferenced to their average and band-pass filtered from 0.5 to 20 Hz—the standard filter settings for this paradigm (Bekinschtein et al. 2009). Each dataset was then converted to SPM12 format (http://www.fil.ion.ucl.ac.uk/spm/) for subsequent analysis. Channels near the neck and eyes were discarded after conversion, since these are often sources of muscle and eye-movement artifacts (36 out of the 129 channels).

#### EEG Source Reconstruction

All analyses focused on the transient evoked by the fifth tone in a quintuple. Accordingly, trials were corrected to a baseline 200 ms before the onset of the fifth tone, which occurred between 400 and 600 ms from trial (i.e., first tone) onset. The time segment used for analysis was –200 ms (from fifth tone) to the end of the trial at 696 ms from fifth tone onset. A group inversion was performed to minimize the variance in source localization (over participants) using MSP—the Bayesian inversion scheme within SPM (Litvak and Friston 2008, Friston et al. 2008) (see also Baillet and Garnero (1997), and López et al. (2014) for technical details). After inversion, three windows of interest were selected—as explained in the next section—to study the sources generating responses to the fifth tone. For each window, images of evoked power (i.e., squared deflection from zero), in source space, were computed for each condition and participant. The General Linear Model (GLM) was used to test for significant effects of the four conditions at each source. The ensuing statistical parametric maps (SPMs) in source space were corrected for multiple comparisons using Random Field Theory in the usual way.

In brief, the source reconstruction proceeds in three steps:

- A coregistration step over participants, which maps sensor-coordinates to source space (dipole) coordinates.
- A forward model for the transformation matrix (Lead-field) from dipole activity to scalp activity.
- The inverse transformation, which optimizes the solution at the source level to best explain scalp data (in terms of sparse sources).

Coregistration between the scalp level and the source level in MRI space was applied. Coordinates at the scalp level were based on a standard GSN-Hydrocel template with 128 channels. Three fiducials were used to map the coordinates from sensor space to source space: nasion, left periauricular point and right periauricular point. A template head model based on the MNI brain was applied with a cortical template mesh of 8196 dipoles, which contains the coordinates of the dipole sources.

As previously stated, we discarded 36 electrodes on the face, neck and cheek from the montage, since they were noisy and dominated by muscle artifacts. This left us with incomplete coverage, making the localization problem more difficult. As a result, we constrained the cortical mesh by selecting cortical areas that are, a priori, most likely to generate evoked responses; that is, only regions of the temporal, frontal and parietal lobes were included for source reconstruction. Figures 9 and 10 and supporting text in the Supplementary Material provide a detailed justification for this choice. We did not include deep sources or sources in the occipital and motor cortices, as there is no prior evidence suggesting that these regions are related to the effects of interest in the present study.

The forward model was computed using the boundary element method (Phillips et al. 2007) as standard in SPM12 for EEG-based source reconstruction, with a three layer head model, that is, skin, skull, and brain. At the source level, a mesh based on an MRI template is used to simulate the 1484 dipolar amplitudes in the brain.

We selected a time-frequency window for the source reconstruction. The frequency band used was the same as used in preprocessing with a range of [0.5 20] Hz. The window used for the source reconstruction is from 400 ms after the first stimulus onset in a quintuple, to the end of the analyzed segment. This included the baseline and the evoked response associated with the fifth tone up to the end of the quintuple.

#### Window Placement for Image Extraction

After source inversion, two analyses were performed in order to characterize the spatial and temporal responses. The first used statistical parametric mapping to test for differences in evoked responses in all sources. The second analysis focused on responses in regions of interest (ROI) based on regionally specific time-series at the source level. The ROIs were defined based upon effects identified by the statistical parametric mapping, as explained below.

Statistical parametric mapping summarizes the activity on the mesh, across the time window chosen. The evoked power was averaged within time windows for the frequency range [0.5 20] Hz. A spatial filter (FWHM = 1 mm) was used to smooth the dipole activity in 3D source space.

Evoked power under each condition was calculated as the root-mean-squared response (in source space) over the window. Statistical inference in this context, then, required the placement of time windows. Tailoring such windows post hoc to the EEG data risks biased sampling and could inflate false positive rates (e.g., Brooks et al. 2017). Consequently, prior precedents for these placements are used. These were taken from Bekinschtein et al. (2009), which introduced the local-global experimental paradigm (see section “Window Placement in the Supplementary Material for further justification of these window settings). Specifically, we looked at the following three effects:

- Early window [100 150] ms: to quantify the local effect, which would be expected to correspond to the MMN in electrode space.
- Middle window [250 350] ms: to assess the interaction between the local and global effects, which is most likely to occur when both local and global effects are present.
- Late window [400 600] ms: to quantify the global effect, which would be expected to correspond to a P3b in electrode space.

## Statistical Analysis

### Hierarchical Spatial Responses

For each participant (18) and each condition (8), three images of evoked power (one for each time window) were created for GLM statistical analysis. The experimental design can be summarized as a 2 × 2 × 2 within-subjects design, with three factors: sedation, local, and global. Each factor comprises two levels: sedation and recovery (for sedation); LS and LD (for local) and global standard (GS) and global deviant (GD) (for global). The ensuing statistical analysis can be summarized as follows.

The first issue was to understand the relationship between local and global manipulations. To do so, we first looked at the local effect and the global effect individually and then the local × global interaction. Since the local and global effects have been extensively explored and are well documented in the literature, our analyses of these effects serve as sanity checks of our source localization. That is, if the MSP algorithm localizes these effects to the expected brain areas, we can have confidence that the reconstruction scheme can localize the effects for which there are fewer precedents.

The second issue was to understand the effect of sedation. We therefore assessed the main effect of sedation, the sedation × local interaction, the sedation × global interaction and the three-way (sedation × local × global) interaction.

We conducted a flexible ANOVA analysis with pooled variance (see Supplementary Material section “Pooled Variance”), which employs a two-step threshold to control for multiple comparisons (Friston et al. 2007). The first level cluster-forming threshold used an uncorrected alpha level of 0.001 to define clusters of voxels. Secondly, random field theory was used to determine the likelihood that a cluster of voxels of a particular size will arise under the null hypothesis. The (cluster level) threshold was a family-wise error (FWE) corrected alpha level of 0.05. This choice of thresholds provides robust protection against false positive rates (Flandin and Friston 2016).

### Temporal Dimension

Source analysis reveals which cortical sources exhibit significant effects of interest, within the different time windows. For illustrative (but not statistical) purposes, we investigated how activity changes through time at the source level, as follows. A source in each brain region—selected to plot the source time-series—was taken from the peak of the significant cluster, during the middle window [250, 350] ms. Specifically, we selected the temporal lobe sources located at the peak of the cluster for the local effect, the frontal lobe sources located at the peak of the cluster in the local × global interaction, and the parietal sources located at the peak of the cluster for the global effect.

The sources were then identified within an ROI of 5 mm, and the corresponding time-series were exported for each subject and each condition. Since statistical results are not being reported on these time-series, issues of double dipping do not arise.

Only the left hemisphere time-series are presented for illustration (the time course for the right hemisphere was very similar). The regional time-series data correspond to the group average of the time-series for each window; namely, the early window [50 100] ms, the middle window [250 350] ms and the late window [400 600] ms. The regional time-series were then preprocessed to provide a quantitative characterization of effect sizes. The regional time-series were smoothed with an adapting hamming window: the points up to the end of the early window were smoothed with a hamming window width of 50 ms. The length of the hamming window then increased linearly from 50 to 100 ms up to the beginning of the middle window. Then, the window size remained the same until the end of the middle window. To deal with the second transition period, the size of the hamming window increased linearly from 100 to 200 ms until the beginning of the late window. From the beginning of the late window onward, the hamming window length was fixed at 200 ms. (This adapting hamming window ensures that the effect size at the center of each window is the value that is entered into the SPM inference). Finally, the root mean square was taken over the source time-series within each ROI. To deal with edge effects, the data were mirrored in time at the beginning and the end of the epoch.

## Results

In this section, the results of the local effect, the global effect, the local-by-global interaction and the three way interaction are presented. Note that the condition-specific time-series presented in this section are positive at all points; that is, they report evoked power (i.e., root mean square responses).

### Local Effect

**Figure 2:**
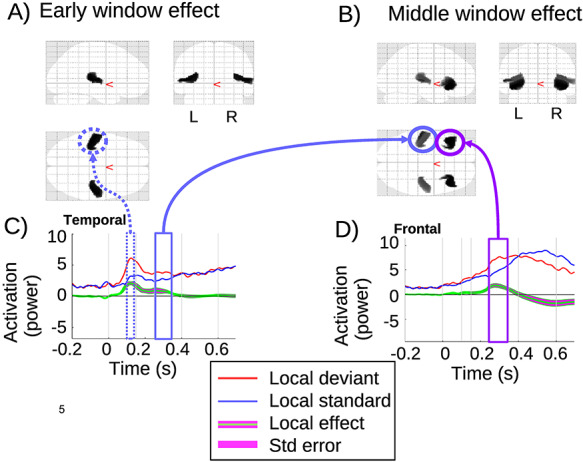
Local effect: (*A*,*B*) present the SPM results with the significant clusters in a 3D glass brain image for the early (*A*) and middle (*B*) windows. (*C*,*D*) The source time-series are plotted for the clusters in the temporal (*C*) and frontal (*D*) lobes. Zero is the onset of the (critical) fifth tone. The time-series are summarized across subjects and shown in red and blue for LD and LS, respectively. The local effect between the two conditions is plotted in green and the standard error in magenta.

The local effect (green line) is significant in the temporal sources, during both the early and middle windows, as shown in Figure 2*A*,*B*. The time course for the temporal area is shown in Figure 2*C* where the MMN appears clearly with a peak in the early window (dashed blue arrow). The local effect is again significant in the temporal region during the middle window (solid blue arrow), whereas it is not significant in the late window. Figure 2*B* shows that frontal clusters are also significant in the middle window. This is shown by the time course of the frontal cluster in Figure 2*D*, with an effect peak during the middle window. Table 1 summarizes the statistical results for each cluster in the early and middle windows. For each cluster, the peak location is described in the second column and the *F*-statistic for the peak of the cluster in the third column. The FWE corrected *P*-value is presented (fourth columns) with the size of the cluster (fifth columns). This shows a strong effect (*P*_FWE_ < 0.001) for all the clusters shown in the table.

**Table 1:** Statistics for each cluster for the local effect in the early and middle windows. Each cluster, named in the first column, is characterized by its peak location in MNI coordinates as shown in the second column, the *F*-value for that peak (third column), the *P*-value (fourth column) and the cluster size (last column). The *P*-value highlights the significant cluster after family-wise error correction, set to an alpha of 0.05

Clusters	Peak location (*x*, *y*, *z*)	*F*[1,119]	*P*_(FWE)_	*K* cluster size
*Early window*				
Left temporal	(−58, −24,4)	15.24	<10^−3^	383
Right temporal	(52, −24,4)	15.14	10^−3^	312
*Middle window*				
Left temporal	(−58, −24, 4)	21.02	<10^−3^	491
Right temporal	(42, −20, 8)	18.9	<10^−3^	365
Left frontal	(−46, 20, −10)	22.65	<10^−3^	591
Right frontal	(−44, 22, −12)	22.59	<10^−3^	584

#### Global Effect

**Figure 3:**
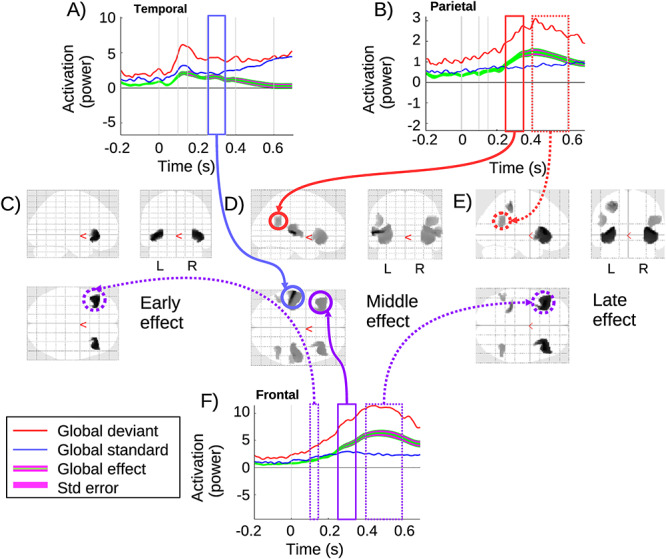
Global effect: (*A*) source time-series corresponding to the temporal lobe cluster, with significant effect in the middle window; (*B*) source time-series corresponding to the parietal cluster, with significant effect in middle and late windows. (*C*,*D*,*E*) 3D glass brain images with significant clusters in early, middle and late windows. (*F*) Source time-series at the frontal cluster, with significant clusters in all three windows. Zero is the onset of the (critical) fifth tone.

The global effect is presented in Figure 3. In the early window, the global effect is significant in both left and right frontal sources, as shown in Figure 3*C*. This early global effect can be related to the contingent negative variation (CNV) (Chennu et al. 2013), which is usually observed at frontal electrodes. Accordingly, the time course for the frontal area in Figure 3*F* shows that the global deviant is greater than the global standard before and during the early window. During the baseline, a small global effect (before the onset of the fifth tone) is also consistent with this CNV effect, as an anticipation of the global deviant quintuple. The nature and implications of this CNV effect are discussed in Section S6 in the Supplementary Material of Shirazibeheshti et al. (2018).

During the middle window, as shown in Figure 3*D*, the global effect is significant in the temporal, parietal, and frontal regions with the time course represented respectively in Figure 3*A*,*B*,*F*. Consistent with Wacongne et al. 2011 and Chennu et al. 2013, this window involves a broad network of brain activity indicated by the significant clusters. Figure 3*A* shows the time course in the temporal area. The global deviant in this area diverges from global standard but is significant only during the middle window, before disappearing in the late window.

In the late window, Figure 3*E*, the global effect is significant in a network comprising both frontal and parietal regions. The time course for the parietal cluster is plotted in Figure 3*B*, which shows that the global effect is significant during the middle and late windows with a peak at the beginning of the late window. Additionally, specifically on the left side, a second more dorsal parietal cluster appears which was not present in the middle window. Finally, Figure 3*F* shows the frontal time course of the global effect, which is significant in all three windows, with a peak in the middle of the late window. This frontal area is the most activated for the global effect, starting from the CNV, until achieving the strongest effect during the late window.

The statistical results from SPM for each significant cluster are shown in Table 2 below. The clusters in the early window have *P*-values (FWE-corrected) of approximately 0.01, while the strongest effects appear during the middle window, with *P*-values (FWE-corrected) below 0.001.

**Table 2:** Statistics for each cluster for the global effect in the early, middle and late windows. Each cluster, named in the first column, is characterized by its peak location in MNI coordinates, as shown in the second column, the *F*-value of the peak (third column), the *P*-value (fourth column) and the cluster size (last column). The *P*-value highlights the significant cluster after family-wise error correction, set to an alpha of 0.05

Clusters	Peak location (*x*, *y*, *z*)	*F*[1,119]	*P*_(FWE)_	*K* cluster size
*Early window*				
Left frontal	(−40, 32, −4)	18.05	10^−2^	177
Right frontal	(44, 26, −6)	17.77	1.3 × 10^−2^	165
*Middle window*				
Left temporal	(−60, −24, 4)	53.88	<10^−3^	682
Right temporal	(52, −24, 4)	31.28	<10^−3^	561
Left frontal	(−46, −20, 10)	28.54	<10^−3^	669
Right frontal	(44, 22, −12)	28.21	<10^−3^	651
Left parietal	(−44, −50, 26)	22.62	<10^−3^	126
Right parietal	(38, −54, 26)	24.92	<10^−3^	265
*Late window*				
Left frontal	(−36, 26, 4)	37.50	<10^−3^	847
Right frontal	(44, 26, 2)	38.32	<10^−3^	822
Right parietal	(40, −50, 24)	20.37	3 × 10^−3^	281
Left parietal 1	(−28, −38, 52)	27.51	3.3 × 10^−2^	158
Left parietal 2	(−46, −50, 22)	19.44	4.8 × 10^−2^	140

#### Local-by-Global Interaction

**Figure 4:**
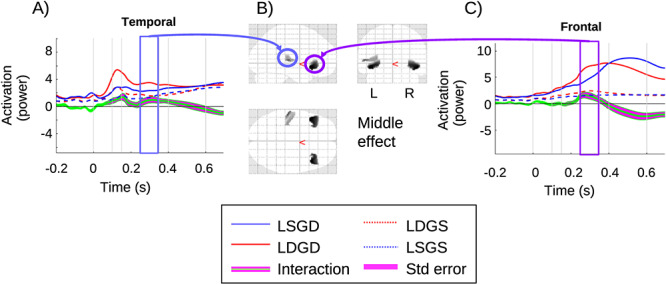
Local-by-global interaction: (*A*) source time-series at the temporal cluster, which is significant in the middle window; (*B*) glass brain of significant clusters for the middle window; (*C*) source time-series at the frontal cluster, which is significant in the middle window. Zero is the onset of the (critical) fifth tone.

The local-by-global interaction is significant only in the middle window, as shown in Figure 4*B*. The left temporal time course is shown in Figure 4*A*, with a small nonsignificant increase in the interaction effect (green line), which peaks after the early window. This is followed by a significant (*P*_FWE_ = 0.003) second increase that is in the middle window. Clusters are also observed in the frontal area. Figure 4*C* shows the time course for the left frontal cluster. The interaction is significant in the middle window (*P*_FWE_ = 0.009), with a positive interaction before a reversal of the effect (green line) in the late window, which does not reach significance. The details of the statistical results from SPM are presented in Table 3. This interaction in the middle window suggests that a frontotemporal network is responsible for linking the local and global.

**Table 3:** Statistics for each cluster for the local x global effect in the middle window. Each cluster, named in the first column, is characterized by its peak location in MNI coordinates as shown in the second column, the *F*-value at the peak (third column), the *P*-value (fourth column) and the cluster size (last column). The *P*-value highlights the significant cluster after family-wise error correction, set to an alpha of 0.05

Clusters	Peak location (*x*, *y*, *z*)	*F*[1,119]	*P*_(FWE)_	*K* cluster size
*Middle window*				
Left frontal	(−46, 20, −10)	13.11	3 × 10^−3^	244
Right frontal	(44, 22, −12)	13.02	3 × 10^−3^	234
Left temporal	(−46, 20, −10)	12.45	9 × 10^−3^	186

#### Three-Way Interaction

**Figure 5:**
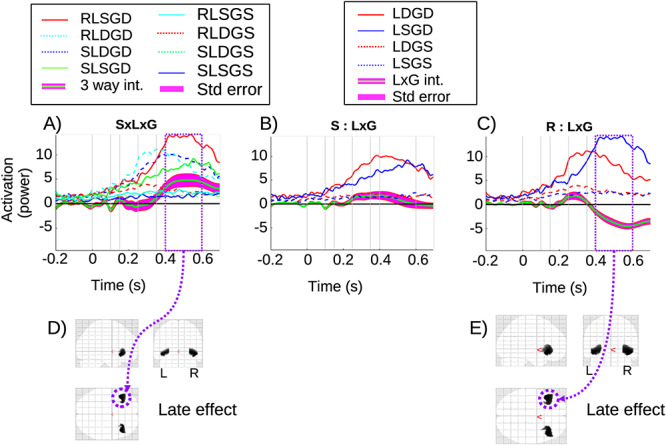
Three-way interaction: (*A*) source time-series for the frontal left cluster of the three -way interaction and the eight conditions involved. (*B*) Local-by-global interaction source time-series for the sedation conditions in frontal cluster. (*C*) Local-by-global interaction source time-series for the recovery conditions in frontal cluster. (*D*) 3D glass brain of the significant clusters for the three-way interaction in the late window. (*E*) 3D glass brain of the significant cluster for the local-by-global interaction when recovered.

Finally, the time-series for all conditions and the three-way interaction (local-by-global-by-sedation) with its standard error are shown in Figure 5*A*. The three-way interaction is significant in the late window, with its corresponding significant clusters in the frontal lobe shown in Figure 5*D*. To characterize the causes of the three way interaction, we explored the two simple effects of interactions that constitute it. Specifically, the local-by-global interaction is presented separately for sedation and recovery in Figure 5*B*,*C*, respectively. Notably, the local-by-global interaction was significant in the late window when participants had recovered, but not when they were sedated. Indeed, the local-by-global effect (green line) had opposite polarities when sedated and recovered for much of the late window. This difference between sedated and recovered seems to be carried by two properties. Firstly, the LDGD condition terminates more sharply when recovered, and secondly, the LSGD condition has a dramatically higher amplitude when recovered. The former is exactly consistent with the deceleration of the accelerated prediction error reported in Shirazibeheshti et al. (2018), suggesting that inferior frontal regions are the source of this shifting neural responsiveness.

The latter of these properties (amplitude increase for LSGD) is particularly striking, and important, since the LSGD condition is—in a sense—the most cognitively demanding condition. In particular, there is no bottom-up deviance (as there is in the LDGD condition) signaling an infringement of global regularity. Thus, higher levels in the processing hierarchy effectively need to detect global deviance by the absence of a driving bottom-up prediction error. Our findings suggest that this capacity is realized by inferior frontal regions, consistent with the often discussed role of prefrontal regions in working memory maintenance and update (Polich 2007). Figure 5*E* shows the significant clusters for the local-by-global interaction (recovery) in the late window. Statistical results in the late window for the three way interaction and for the recovery local-by-global simple effect are presented in Table 4, all *P*-values are highly significant.

**Table 4:** Statistics of three-way interaction for both frontal clusters in the late window and local-by-global interaction for recovery. Each cluster, named in the first column, is characterized by its peak location in MNI coordinates, as shown in the second column, the *F*-value of the peak (third column), the *P*-value (fourth column) and the cluster size (last column). The *P*-value highlights the significant cluster after family-wise error correction, set to an alpha of 0.05

Cluster	Peak location (*x*,*y*,*z*)	*F*[1,119]	*P*_(FWE)_	*K* cluster size
*Three-way interaction: late window*				
Left frontal	(−46, 20, −10)	12.21	5 × 10^−3^	267
Right frontal	(46, 22, −10)	12.22	5 × 10^−3^	261
*L × G int. for recovery: late window*				
Left frontal	(−46, −20, −10)	17.09	<10^−3^	516
Right frontal	(44, 22, −12)	17.24	<10^−3^	519

Additionally, we found a significant effect of sedation in the temporal region, and a significant sedation-by-local interaction in the frontal region, which are presented in the Supplementary Material (section “Further Sedation Effects”). The sedation-by-global interaction was not found to be significant.

## Discussion

We have presented a source localization of the neuronal responses evoked by the local-global paradigm—and the effect of sedation with propofol on these responses. In this way, we have addressed how two key cognitive neuroscience theories—PC and the GW—are related and the neurophysiological correlates of this interrelationship.

Importantly, the two theories (PC and GNW) do already include some related concepts. In particular, prediction has been discussed within the GNW context, for example, the original formulation of GNW (Dehaene et al. 1998) did discuss “anticipation” and prerepresentation. Additionally, and perhaps most notably, Wacongne et al. (2012) presents an important neural instantiation of predictive processing, which simulates the mismatch-negativity. However, we would argue that PC goes further, by providing a full multilevel architecture of brain processing that is theoretically grounded in the mathematics of (generative) Bayesian inference (Friston 2010), with concepts such as confidence in (i.e., the precision of) the prediction error to the fore. In this regard, PC provides a full instantiation of the core hypothesis that the cortical architecture is configured to minimize the difference between bottom-up representations, driven from sensory input, and top-down representations of expectations, with this interpretation obtaining at all hierarchical levels. In this sense, there is a clear need to reconcile PC and the GNW.

In respect of neural correlates, it is important to acknowledge the constraints associated with our source localization analysis. In particular, we explicitly placed masks (which were justified by prior precedent; c.f. Supplementary Material section “Subspace selection and mask placement”) to constrain the source reconstruction. In this respect, it is not surprising that we found sources in these a priori regions. However, exactly where those sources fell, especially in the large regions of the frontal and parietal masks, is of interest. Of greater note is how the MSP algorithm unmixed variability among the regions and how that unmixing progresses through the time-course of the evoked response. In this respect, a sanity check of our findings is that, as one would expect, early effects are temporal, with a following propagation out from this sensory region to frontal and parietal regions.

The local and global main effects we observed also have considerable face validity. In particular, as shown in Figure 2*A*, the local effect is detected exclusively in temporal sources in the early window, consistent with sources reflecting the MMN in auditory cortices (Naatanen et al. 2007). The local effect then engages a (inferior) frontal—(superior) temporal network in the middle window, see Figure 2*B*, which corresponds to the source of the positive rebound to the MMN (Naatanen et al. 2007). This rebound is often related to the P3a, which is known to be generated by frontal sources (Polich 2007).

The classic global effect pattern is apparent in the middle and late windows, with what could be considered a prototypical GW pattern involving (inferior) frontal—(superior) temporal and parietal regions. Importantly, parietal sources are only present in this contrast, suggesting a unique role for parietal regions in the GW. Additionally, there is a clear progression during the global effect from middle to late windows, in which the temporal source wanes, while frontal and parietal sources wax. This suggests a trajectory over time of the GW from sensory to encompass association regions. We can highlight four key findings of our analysis:

1. three phases of processing (early, transitional, and late);
2. frontotemporal interaction between local and global in the transitional phase;
3. a failure to detect a main effect of sedation in parietal sources; and
4. a three-way interaction between propofol and local and global effects

We elaborate on these in turn.

**Figure 6:**
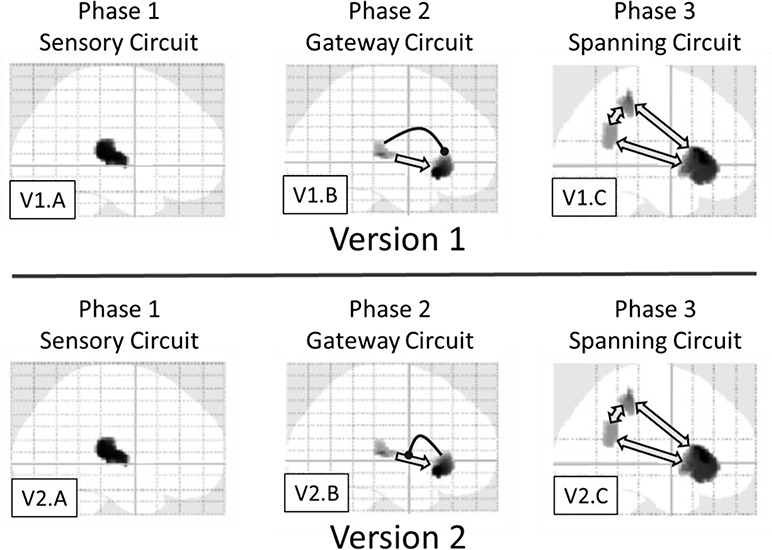
Depiction of three-phase theory of local-global processing. Two different versions are presented, which are distinguished by the direction of modulatory activity (c.f. link with circle at end). In both versions, phase 1 (c.f. V1.A and V2.A) is restricted to sensory areas, reflecting a sensory prediction error; phase 2 (c.f. V1.B and V2.B) involves interaction between sensory and an inferior frontal region; and phase 3 (c.f. V1.C and V2.C) involves a deep, brain-scale hierarchy, analogous to activation of a GW. Importantly, we hypothesize that phase 2 is the transition between phase 1 and phase 3, effectively regulating “ignition” of the GW, according perhaps to the precision or priority afforded by the ascending (prediction error) signal. The difference between the two versions presented here, with direction of phase two modulation being key, is elaborated in the discussion.

1) Three phases: Figure 6 depicts the neurophysiological realizations of the putative three phases. As discussed previously, the local effect manifests in the early window (early phase) in source space, very much as one would expect—expressed predominantly in superior temporal regions, which include auditory cortices. Additionally, a stereotypical GW is present in the late window (late phase). Importantly, our middle window (transitional phase) appears to exhibit qualitatively distinct effects, in terms of the set of sources involved and condition-specific effects exhibited. In particular, the local and global effects only interact in the middle window, suggesting a modulatory exchange between temporal and inferior frontal regions, where the local-by-global interaction was expressed.

With a PC and GW perspective in mind, one could argue that the three phases can be distinguished according to the following characteristics.
Phase 1 (sensory): activation is restricted to (auditory sensory and perceptual) superior-temporal regions. This phase is expressed over a short time frame, with key responses being consistent with the forward propagation of a bottom-up (sensory) prediction error.
Phase 2 (gateway/transitional): this phase can be hypothesized to engage a mesoscale network (similar to that considered in Phillips et al. 2015, 2016 for the MMN), involving an exchange between (auditory) superior-temporal and inferior-frontal sources. The network features modulatory dynamics, which may implement a priority-based or precision enhancement—consistent with the attentional selection of precise prediction errors. This gateway circuit exhibits a more sustained phasic response than phase 1, but is not metastable in the sense of phase 3.
Phase 3 (spanning): this circuit is argued to be macro/brain-scale, spanning the cortical hierarchy. It would naturally be related to the GW (Dehaene 2011) and would exhibit a prolonged, metastable response, as discussed in King et al. (2014).

2) Local times global interaction and the transition phase: to elaborate further, the interaction between local and global in the middle window suggests a multiplicative or modulatory exchange between superior-temporal regions and inferior frontal areas. The former of these elaborates sensory prediction errors that underlie the local effect, and the latter is implicated in higher order processing that integrates over a longer temporal scale.

This network may effectively be a subcircuit of the brain spanning GW, mediating a transitional state, indeed perhaps a proto-workspace. Although further work is certainly required to confirm the hypothesis, it could be argued that the local-by-global interaction in the middle window is suggestive of bidirectional exchanges between levels in a predictive hierarchy, with the multiplicative interaction between levels suggestive of modulation of gain control, which, under PC, could be generated by predictions of precision (Kanai et al. 2015).
The Monkey ECoG findings of Chao et al. (2018) sit well with this interpretation. Most importantly, Chao et al. (2018) also highlight a mesoscale circuit between superior temporal areas and prefrontal areas, associated with hierarchical predictive processing. Given the spatial resolution of the source localization we have performed, there is good alignment between the localizations, with (1) our superior temporal source encompassing both the auditory cortex and anterior temporal sources of Chao et al. (2018); and (2) the prefrontal sources of the two studies intersecting (note, monkey prefrontal cortex is anterior and considerably smaller than human prefrontal cortex). Additionally, the dynamic causal modeling work on the omission of an expected stimulus (Chennu et al. 2016a) also points to a temporofrontal circuit with strong feedback influences.

3) No parietal sedation effects: given the statistical power available, we did not detect any effects of sedation on parietal areas—while strong effects were found frontotemporally. This stands against an existing finding of propofol-induced modulation of parietal networks (Schrouff et al. 2011; Uhrig et al. 2016), which we discuss further shortly. From a GW perspective, this might seem surprising, since the parietal cortex has been argued to be implicated in conscious experience. This said, with classical statistics, null effects are always difficult to interpret (Ideally, one would like to perform a Bayesian analysis to find evidence for the null. However, it is not currently clear how to formulate a Bayes Factor that accurately reflects the inferential steps involved in a family-wise error corrected neuroimaging analysis.), and there remains the possibility that a more highly powered experiment would find an effect at parietal lobe.

Inference though can be less equivocal with regard to the significant effects involving the sedation factor. In particular, we observed a temporal source for the main effect of sedation, see Figure 7*A*,*B* of the Supplementary Material, which, in the early window, is consistent with the known enhanced N1 in anesthesia (Yppärilä et al. 2004) but for us was also seen in the middle window. Additionally, temporal and frontal sources evidenced a sedation × local interaction; see Figure 7*E*,*F* of Supplementary Material. This provides some evidence that sedation modulates responses early in the processing pathway. The most interesting effect though was the local by global by sedation effect, which was significant in the late window.

4) Three-way interaction: this was observed at an inferior frontal source; see Figure 5. When we decompose this three-way interaction into its component two-way simple effects—local-by-global when recovered and when sedated—the cause of the three-way is clear. There are two particular aspects to emphasize.

Firstly, the acceleration of the global deviant response by the coincidence of local deviance (the double surprise acceleration effect (Shirazibeheshti et al. 2018) is evident at the inferior frontal source both when sedated and when recovered, with this acceleration being stronger when recovered. This is apparent in the sharper onset and offset of the LDGD condition when recovered compared with sedated (see Fig. 5*B*,*C*). This suggests that the deceleration of the accelerated prediction error described in Shirazibeheshti et al. (2018), can be localized to inferior frontal regions.
Secondly, the most striking feature driving the three way interaction at frontal in the late window is the dramatically higher LSGD condition when recovered than when sedated (see Fig. 5*B*,*C*). Importantly, the LSGD condition is most dependent upon long-term temporal integration. In particular, this global deviance is not marked by a sensory prediction area (since it arises during a LS quintuple). Thus, the deviance is not initiated by a strong bottom-up signal (i.e., a local prediction error). That is, it is a pure global deviance condition, with its detection intrinsic to higher hierarchical levels.
It seems then that sedation impairs this capacity to detect deviance intrinsically at higher levels, at least at inferior frontal sources. This finding is in many respects consistent with the intent of the local-global task; that is, to differentiate processing that requires temporally sustained integration over an extended period of time, and the role of consciousness in this temporally extended evidence accumulation. Thus, our findings provide suggestive evidence that, in respect of the action of propofol, reduced awareness diminishes long duration processing of temporal integration, supported by inferior frontal sources. In terms of PC, this finding is consistent with a reduction in the precision of ascending prediction errors. This follows because precision corresponds to the rate of evidence accumulation (Hesselmann et al. 2010; FitzGerald et al. 2015). In other words, belief updating in response to precise prediction errors converges more quickly than in the setting of imprecise prediction errors—or a pharmacological reduction in gain of responsiveness of populations encoding prediction errors. This particular effect of propofol would endorse the hypothesis we highlighted earlier that ignition depends upon a phase transition that itself rests upon attentional selection of ascending prediction errors that is mediated by the modulatory effects of predicted precision.

### Sedation

The “sedation state” that we are exploring involves placing the participant at the fringe of consciousness, which may correspond to a (weakened, but) active bottom-up stimulus strength and lower top-down attention. In this regard, it may be comparable with the “preconscious” state in (Dehaene et al. 2006). However, it is notable that in our data, sedation reduces, although seems not to eliminate, the sensory response, for example, see Supplementary Material section “Further Sedation Effects,” where Figure 7*A*–*C* shows a clear sedation effect at sensory areas. Because event-related potentials are averages across many trials, it is always difficult to know whether the reduction of a component is due to a consistent reduction at the single-trial level, or increased variability in the response. Thus, it is possible that the reduction in sensory components with sedation that we observe are caused by intermittent activation, in which sensory input is extremely weak, even absent, on some trials, and strong, presumably with the GW fully engaged, on others. Resolving these competing explanations awaits further investigations.

Our findings on the effect of propofol sedation resonate with a number of previous findings. Firstly, Boly et al. (2012) suggest that patients with reduced levels of consciousness (i.e., vegetative state) exhibited a reduction in effective connectivity for a descending extrinsic (between source) connection from inferior frontal to superior temporal regions. This link was found absent during a MMN paradigm, very similar to the local component of the local-global task. While we are limited in our capacity to decompose our temporal region anatomically, and thus to directly implicate superior temporal regions beyond primary auditory cortex, we have found effects of sedation in (superior) temporal/inferior-frontal regions, although, care should certainly be taken in relating two different forms of reduced awareness (vegetative state and sedation).

Uhrig et al. (2016) present an impressive fMRI study of the effects of anesthesia on brain responses to the local-global effect in monkeys, in which they report a reduction in prefrontal responses to global deviance during sedation. Additionally, Nourski et al. (2018) observed a striking abolition of prefrontal responses to global deviance with sedation. As previously discussed, we observe similar effects, although our findings are within the context of our local-by-global interaction enabling us to clarify the nonadditive relationships between local and global levels. Additionally, Uhrig et al observed an effect of sedation on the local effect, which is consistent with the local × sedation interaction we report in the Supplementary Material (subsection “Further Sedation Effects”).

Where there is some inconsistency with our findings is in respect of parietal sources. Uhrig et al. (2016) identified reduced global deviant responses parietally with anesthesia; however, we failed to find any significant interaction effects involving sedation and global at parietal sources. A failure to reject the null does not, of course, enable its affirmation, leaving this question not definitively answered. Although, it should be noted that, (in a psycho-physiological interaction) for the global effect, Uhrig et al. (2016) did observe a residual context-dependent coupling between auditory areas and intraparietal sulcus during moderate propofol sedation, suggesting that, even in their study, sedation did not fully obliterate parietal responses for the global effect.

In summary, although there remains considerable uncertainty, the key ERP components we observe are (increased) responses to unexpected/deviant stimuli, which would naturally be considered a signaling of prediction error. In this context, we can give a more specific candidate interpretation of the effects of sedation: PC suggests that the feed-forward error signal reflects a prediction error weighted by its precision or confidence afforded that error signal. Confidence in our context would be driven by an assessment of the level of irreducible sensory prediction error—or by descending predictions of precision based upon belief updating higher in the hierarchy (see Fig. 6). The two key effects of sedation that we observe are (1) a reduction in amplitude and (2) a slowing of the evoked response to deviant stimuli, which are particularly pronounced with global deviance. Indeed, this is marked in the evoked response to the simultaneous confounding of expectations at multiple hierarchical levels; namely local and global. A likely candidate for both of these reductions—in amplitude and in speed—would be reduction of gain/precision (Kanai et al. 2015), which would induce a broad loss of responsiveness.

### Top-Down or Bottom-Up Ignition?

Our source localization implicates a superior-temporal—inferior-frontal circuit in this modulation of response by sedation. Although—on the basis of the findings presented here—we cannot be certain of the direction of modulatory influence (descending or ascending) in this circuit. This is reflected in the two versions of the three phase theory presented in Figure 6.

Shirazibeheshti et al. (2018) proposed that the acceleration of the global response—due to coincidental local deviance—is caused by a feed-forward modulation from the local effect circuit onto the global effect circuit. This is the direction of modulatory influence presented in Figure 6, version 1, see panel V.1B. This might be considered an explanation of the acceleration by double surprise that sits most easily with the simplest line of temporal causation, since registration of local deviance would naturally be considered to precede registration of global deviance.

However, the other direction cannot be excluded. That is, it could be that a weakening of the modulatory influence of inferior-frontal on superior-temporal areas is what drives the slowed and attenuated responses observed when sedated. Such an explanation would be consistent with the second version of our three-phase theory presented in Figure 6, where modulation is mediated in a feedback direction (i.e., descending predictions of precision), see panel V2.B. From a PC perspective, as previously suggested, a potential explanation of the effect of sedation is that it reduces the precision of sensory prediction errors, potentially carried by a feedback link from inferior frontal to temporal regions, as per Figure 6 (V2.B). This would effectively attenuate the gain on the ascending transmission of prediction errors. As noted in the introduction, this formulation of the transition from local to global processing (i.e., ignition of the GW) provides a graceful synthesis of PC and GW theory that is grounded in neurophysiology (via gain control and neuromodulation)—while at the same time speaks to psychological concomitants of conscious processing (via attentional selection that accompanies perceptual synthesis over extended periods of time).

## Acknowledgment

The authors acknowledge funding from UK Engineering and Physical Sciences Research Council (SC, Ref: EP/P033199/1); KJF was funded by a Wellcome Trust Principal Research Fellowship (Ref: 088130/Z/09/Z). The dataset was collected based on a Wellcome Trust Clinical Research Training Fellowship (grant.083660/Z/07/Z) in Cambridge (RA). Finally, we would like to acknowledge funding from the 50 years anniversary of Kent (LL, AW).

## Notes

We would like to thank Guillaume Flandin for very valuable discussions of SPM analyses. *Conflict of Interest*: None declared.

## Supplementary Material

LocalisingTheLocalGlobal_v8_SM_bhaa071
