# Supplementary material for: Sedation Modulates Frontotemporal Predictive Coding Circuits and the Double Surprise Acceleration Effect
Source: Cereb Cortex. 2020 May 19;30(10):5204–17. doi: 10.1093/cercor/bhaa071 (PMC7472187; doi:10.1093/cercor/bhaa071)
Supplement: LocalisingTheLocalGlobal_v8_SM_bhaa071 [file localisingthelocalglobal_v8_sm_bhaa071.docx]

# Supplementary Material

# Further sedation effects

# The sedation effect is significant in the temporal sources both in the early and middle windows, as shown in Fig 7A) and Fig 7B) and Table 5. Fig 7C) shows the time course for the temporal cluster, with the largest effect in the first window.

**
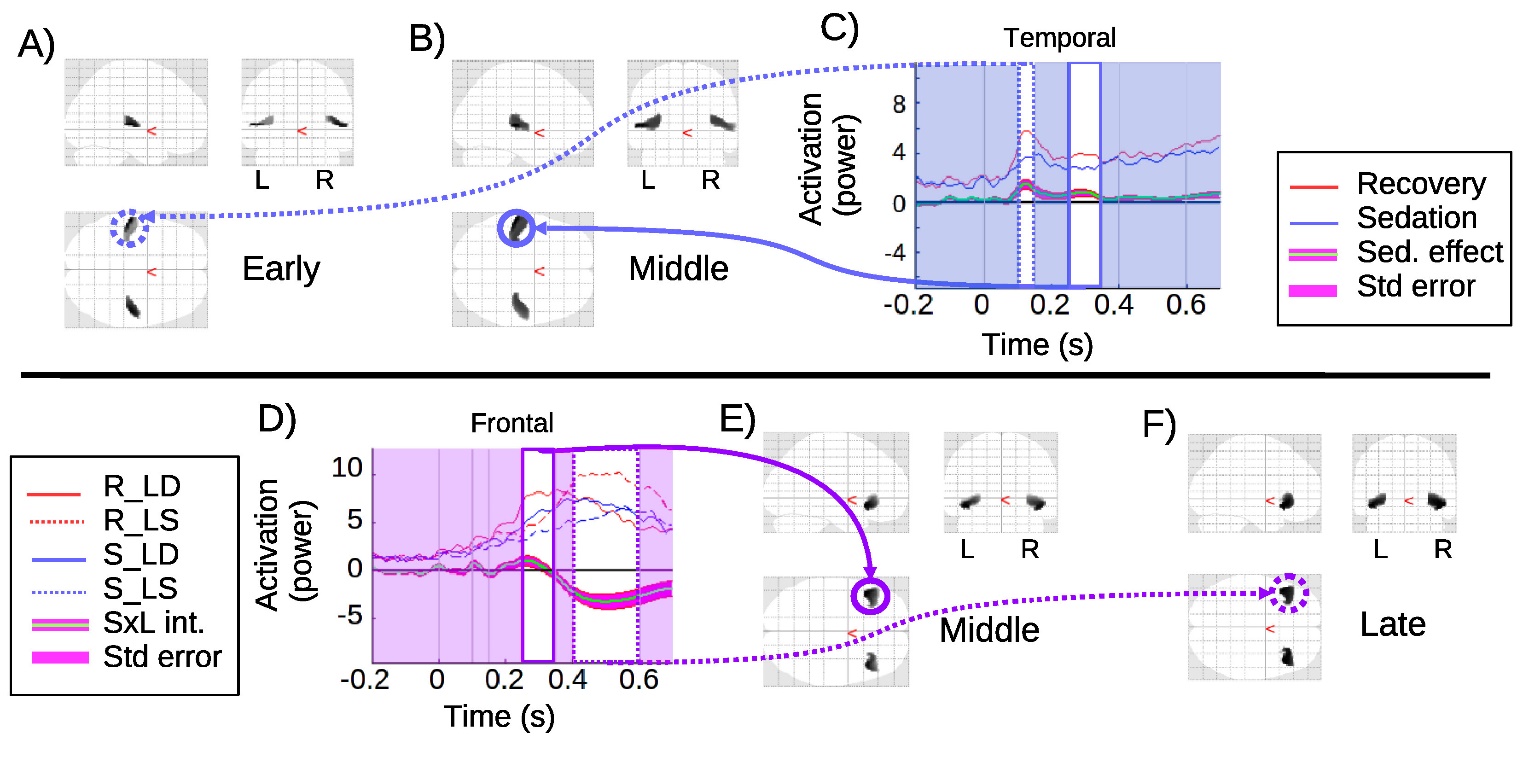
**

1. **Figure 7.** *This figure shows the results of the inversion for the sedation effect (A-C) and for the sedation-by-local interaction (D-F).*

| Sedation effect | | | | |
| --- | --- | --- | --- | --- |
| 1. Cluster | 1. Peak location 2. (x,y,z) | 1. F[1,119] | 1. P_(FWE)_ | 1. K cluster size |
| 1. Early window | | | | |
| 1. Left temporal | 1. (-48, -26, 4) | 1. 21.81 | 1. <1e-3 | 1. 518 |
| 1. Right temporal | 1. (50, -20, 4) | 1. 21.58 | 1. <1e-3 | 1. 408 |
| 1. Middle window | | | | |
| 1. Left temporal | 1. (-46, 24, 4) | 1. 14.79 | 1. <1e-3 | 1. 364 |
| 1. Right temporal | 1. (42, -20, 8) | 1. 13.81 | 1. 0.002 | 1. 269 |

1. **Table 5.** *Statistics of the sedation effect in the early and middle windows. Each cluster, named in the first column, is characterized by its peak location in MNI coordinates as shown in the second column, the F-value of the peak (third column), the p-value (fourth column) and the cluster size (last column).* The p-value highlights the significant cluster after family-wise error correction, set to an alpha of 0.05.
2. The sedation-by-local interaction is significant at both the left and right frontal lobes, as shown in Fig 7E) and F) with the 3D glass brain and Table 6. The time-series for the left frontal cluster is presented in Fig 6D), with a first peak in the middle window and an effect reversal in the late window.

| Sedation-by-local interaction | | | | |
| --- | --- | --- | --- | --- |
| 1. Cluster | 1. Peak location 2. (x,y,z) | 1. F[1,119] | 1. P_(FWE)_ | 1. K cluster size |
| 1. Middle window | | | | |
| 1. Left frontal | 1. (-46, 20, -10) | 1. 12.58 | 1. 0.007 | 1. 199 |
| 1. Right frontal | 1. (44, 22, -12) | 1. 12.48 | 1. 0.009 | 1. 188 |
| 1. Late window | | | | |
| 1. Left frontal | 1. (-46, 20, -10) | 1. 12.08 | 1. 0.007 | 1. 241 |
| 1. Right frontal | 1. (46, 22, -10) | 1. 12.10 | 1. 0.006 | 1. 248 |

**Table 6.** *Statistics of the sedation effect in the middle and late windows. Each cluster, named in the first column, is characterized by his peak location in MNI coordinates as shown in the second column, the F-value for the peak (third column), the p-value (fourth column) and the cluster size (last column).* The p-value highlights the significant cluster after family-wise error correction, set to an alpha of 0.05*.*

## Pooled Variance

# We have employed a pooled, rather than partitioned, variance approach [Friston2007, Chapter 13]. This is because a partitioned approach would require localisation to be performed on contrast maps. This would have brought two issues: (1) contrast data has a lower signal-to-noise ratio, reducing the accuracy of the source localisation, and (2) polarity is lost during MSP localisation, making it unclear how to perform the one-sample t-test in source space that would be required for the final statistical test. Pooling variance avoids the need to apply contrasts in this way, since it only requires simple parameter maps to be available. Penny and Henson [Friston2007] discuss the pros and cons of pooled versus partitioned approaches.

**Window placement**

1. Statistical inference on the results of a Multiple Sparse Priors (MSP) source localisation requires windows to be placed in the evoked time course. To run the second level across participant SPM inference, three dimensional images are required. Since these are in source space, in contrast with SPM inference on the scalp, all three dimensions are spatial. This enables SPM inference to follow the recipe of a standard second-level SPM three dimensional mass-univariate analysis [Penny et al, 2011].
2. To obtain such three dimensional maps from the four dimensions – three of space and one of time – generated by the MSP algorithm, time windows are placed and collapsed across, by calculating the root mean square value across the temporal extent of the window. Statistical inference in this context, then, requires the placement of time windows.
3. Tailoring such windows post hoc to the landmarks of M/EEG data will inflate false positive rates (e.g. [Brooks2017]). Thus, we document here how we have arrived at our window placements, the prior precedents for which are taken from Bekinschtein et al [Bekinschtein 2009], the article that introduced the local-global effect.
4.
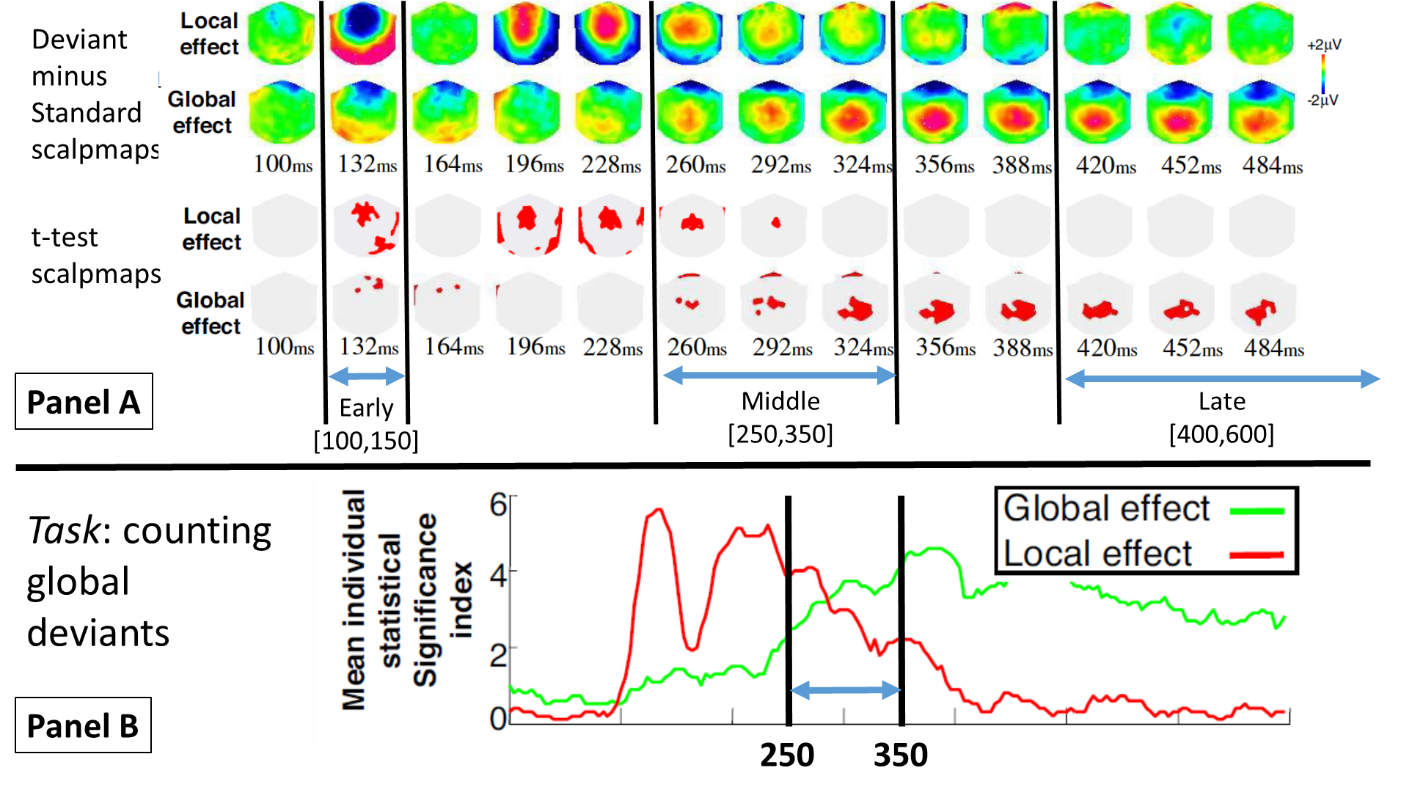

5. **Figure 8.** *Figures from [Bekinschtein 2009], reproduced here to explain our window placements and the prior precedents for them. Panel A is the top part of figure 2 from [Bekinschtein 2009], which shows the basic local and global effects observed. Panel B is part of figure 4 from [Bekinschtein 2009]: it is a counting global deviants condition, which is the task performed in our experiment. The windows we have selected are shown with light blue double arrows.*
6. We place three windows to look at three effects:

- early window: to look at the local effect, i.e. the mismatch negativity;
- middle window: to look at the interaction between the local and global effects, which is most likely to occur when both local and global effects are present;
- late window: to look at the global effect, which manifests as a P3b.

1. We identified relevant scalp maps in [Bekinschtein 2009] Figure 2 (see our Fig 8, panel A), and placed windows between relevant and the first adjacent non-relevant scalp maps.
2. As shown in Fig 8, our local effect window starts at the beginning and finishes at the end of the local effect scalp map in [Bekinschtein 2009].
3. The middle window was selected as the largest period in which both local and global effects are present. Our middle window extends out beyond the 324ms scalp map. This is because Figure 4, top-row, right hand panel of [Bekinschtein 2009], which is reproduced in Fig 8 (panel B), shows that the local effect remains substantial (and the global effect even more so) out to the 350ms region – marked in our Fig 8 (panel B).
4. Our late window has been selected to be sufficiently far from the middle window that we investigate a distinct region of the time series. However, we keep the gap between middle and late windows relatively small (50ms), since, as evident in [Bekinschtein 2009] (reproduced in our Fig 8), the P3b may be waning in size from around 400 ms. We select a relatively long late window, since there is considerable prior precedent for an extended P3b; see, for example, [King2014] and panel B of our Fig 8.

**Figure 9.** *We present figure 3 from Garrido et al, 2008, showing the key MMN sources that they use in their DCM fitting.*

## Subspace selection and mask placement

Our source localisation analyses have used masks to guide the localisation algorithm. We justify the placement of these masks here.

**
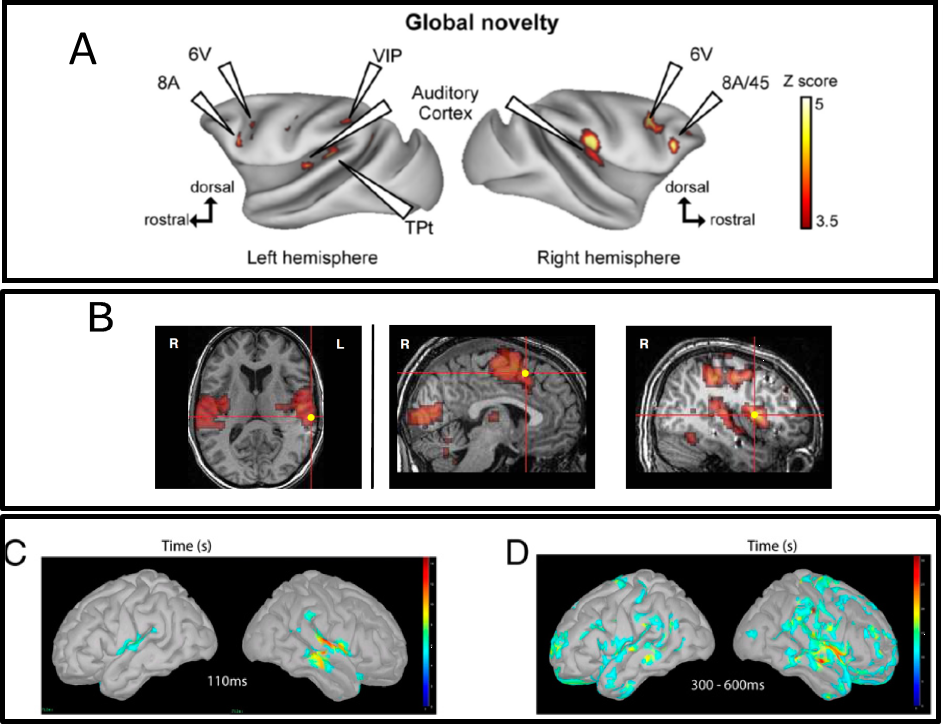
**

**Figure 10.** Precedents for subspace placement. *A) presents panel a) from figure 4 of Uhrig et al, 2014 showing fMRI responses to the global effect in monkeys. Activated brain areas include superior temporal cortex, 8A/45, prefrontal cortex area 8A/45; 6V, premotor area 6V; VIP, ventral intraparietal area; 8A, prefrontal area 8A; TPt, temporoparietal area. These areas have prompted the placement of our masks. B) shows panel (A) of figure 3 of [Bekinschtein2009], which gives the results of an fMRI analysis of the local-global task. The leftmost panel presents areas engaged by the local effect, while the middle and right panels show areas engaged by the global effect. C) and D) are reported from Figure 3 of [Wacongne2011], which show the results of a source localisation of the local-global task. The left panel C) shows the generators of the local effect, while the right panel D) shows the much more broadly dispersed generators of the global effect.*

**Local Effect**

There is considerable previous literature that presents the generators of the mismatch negativity, which corresponds to our local effect. These prior precedents were reflected in the sources used in Garrido et al, 2008, which are presented in Fig 9. Additionally, Chennu et al, 2016 showed that Garrido et al’s mismatch negativity sources also fit the local effect, as it arises in the local-global paradigm.

The four temporal sources shown in Fig 9 (lA1, lSTG, rA1 and rSTG) become combined in our analysis into two temporal sources: one on the left and one on the right. We do not have sufficient temporal resolution to distinguish primary auditory cortex from superior temporal gyrus. The central role of superior temporal regions in the MMN is also consistent with the studies of [Bekinschtein 2009] and [Wacongne2011], see Fig 10B) left most panel and Fig 10C). The Garrido et al work also provides a prior precedent for frontal masks, which include inferior frontal areas, see Fig 9.

**Global Effect**

Localisation precedents for the global effect are somewhat more varied than for the local effect, but nonetheless, there are previous studies that justify placement of broad masks in frontal and parietal regions. Three precedents are shown in Fig 10 panel A), Fig 10 panel B) (middle and right panels) and Fig 10D). As can be seen, a broad range of sources were identified across the cortical sheet, including frontal, parietal, and temporal sources.


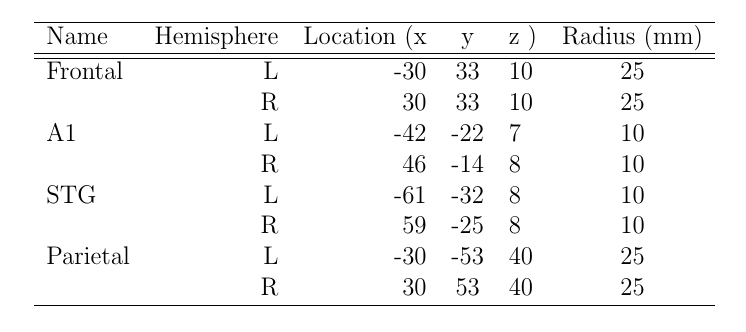
***Table 7.*** *Parameters of the spheres for the masks*

Fig 11 represents the dipoles used for the inversion, as a subspace of the normal mesh. This includes small spheres for the temporal lobes and big spheres for the frontal and parietal lobes. The temporal spheres include the Superior Temporal Gyrus (STG) and the primary auditory cortex, taken from [Garrido2008]. For frontal and parietal, we selected the best location that maximizes the space in the frontal and parietal area without overlapping in the midline. The coordinates for each prior are shown in the Table 7.

**Figure 11.** *Sub-space: glass brain representation of the dipoles used for the source inversion scheme after applying the masks*
